# An Integrated Intervention for Increasing Clinical Nurses’ Knowledge of HIV/AIDS-Related Occupational Safety

**DOI:** 10.3390/ijerph13111094

**Published:** 2016-11-07

**Authors:** Liping He, Zhiyan Lu, Jing Huang, Yiping Zhou, Jian Huang, Yongyi Bi, Jun Li

**Affiliations:** 1 School of Public Health, Wuhan University, Wuhan 430072, China; hlipingm@163.com; 2 School of Public Health, Xiangnan University, Chenzhou 423000, China; xnxyzyp@163.com; 3 Department of Radiology, Zhongnan Hospital of Wuhan University, Wuchang District, Wuhan 430072, China; luzhiyan@whu.edu.cn; 4 The Affiliated Hospital of Xiangnan University, Chenzhou 423000, China; huangjing5093@163.com; 5 Chenzhou City Center for Disease Control and Prevention, Chenzhou 423000, China; Chenzhouhiv06@sina.com

**Keywords:** nurses, occupational exposure, HIV, knowledge, early intervention

## Abstract

*Background*: Approximately 35 new HIV (Human Immunodeficiency Virus, HIV) cases and at least 1000 serious infections are transmitted annually to health care workers. In China, HIV prevalence is increasing and nursing personnel are encountering these individuals more than in the past. Contaminated needle-stick injuries represent a significant occupational burden for nurses. Evidence suggests that nurses in China may not fully understand HIV/AIDS (Acquired immunodeficiency syndrome, AIDS) and HIV-related occupational safety. At this time, universal protection precautions are not strictly implemented in Chinese hospitals. Lack of training may place nurses at risk for occupational exposure to blood-borne pathogens. *Objectives*: To assess the effectiveness of integrated interventions on nurses’ knowledge improvement about reducing the risk of occupationally acquired HIV infection. *Methods*: We audited integrated interventions using 300 questionnaires collected from nurses at the Affiliated Hospital of Xiangnan University, a public polyclinic in Hunan Province. The intervention studied was multifaceted and included appropriate and targeted training content for hospital, department and individual levels. After three months of occupational safety integrated interventions, 234 participants who completed the program were assessed. *Results*: Of the subjects studied, 94.3% (283/300) were injured one or more times by medical sharp instruments or splashed by body fluids in the last year and 95.3% considered their risk of occupational exposure high or very high. After the intervention, awareness of HIV/AIDS-related knowledge improved significantly (*χ*^2^ = 86.34, *p* = 0.00), and correct answers increased from 67.9% to 82.34%. Correct answers regarding risk perception were significantly different between pre-test (54.4%) and post-test (66.6%) (*χ*^2^ = 73.2, *p* = 0.00). When coming into contact with patient body fluids and blood only 24.0% of subjects used gloves regularly. The pre-test knowledge scores on universal precautions were relatively high. Correct answers about universal precautions improved significantly from pre-test (83.71%) to post-test (89.58%; *χ*^2^ = 25.00, *p* = 0.00). After the intervention, nurses’ attitude scores improved significantly from pre-test (3.80 ± 0.79) to post-test (4.06 ± 0.75; *t* = 3.74, *p* = 0.00). *Conclusions*: Integrated educational interventions enhance nurses’ knowledge of risk reduction for occupationally acquired HIV infections and improve the observance of universal precautionary procedures. This enhancement allows nurses to assume a teaching role for prevention and management of HIV/AIDS.

## 1. Introduction

UNAIDS (the Joint United Nations Programme on HIV/AIDS, UNAIDS) and the WHO (World Health Organization, WHO) estimate that approximately 37.0 million people worldwide had HIV (Human Immunodeficiency Virus, HIV) by the end of 2014 [1]. The WHO defines HIV occupational exposure in health care workers as percutaneous injuries (e.g., needle-stick and sharps injuries, NSIs) or the contact of mucous membranes or non-intact skin (chapped, abraded or diseased) with blood, tissue, or other body fluids that are potentially HIV-infectious [2]. The average risk of HIV transmission from a positive source is estimated to be 0.3% by a single percutaneous exposure and 0.09% by mucous membrane exposure. With increasing volumes of blood and viral loads in the exposures, transmission risk is greater [3,4]. In 2003, the WHO reported that from 14 geographical regions, the burden and fraction of HIV infections attributable to needle/sharp instrument injuries of heath care workers was three million, and these led to about 5.5% new HIV cases (range 0.5%–11%) [2]. Nurses represent the greatest proportion of exposed heath care workers as they are frontline providers who more often encounter patients with HIV/AIDS and can be more accessible for AIDS prevention and control. Therefore, nurses are ideally positioned to have a role in HIV/AIDS prevention [5].

In China, 86% of nurses were reportedly injured by needles and splashed with body fluids, and of these subjects studied 59.7% were concerned about contracting HIV/AIDS [6]. In contrast, approximately 50% to 67% of NSIs were unreported [7]. Worse, some studies suggest that many nurses in China still do not fully understand HIV/AIDS transmission pathways. Although universal precautions can protect heath care workers from HIV transmission through occupational exposure [8], 85% of nurses lack universal precautions practices and are often occupationally exposed to HIV in rural China [9]. Moreover, in China, studies about HIV/AIDS occupational exposures depend on officially reported data and thus few interventions have been undertaken [10,11] and nurses’ roles in HIV/AIDS prevention and management are less recognized [12].

Therefore, we studied the incidence of NSIs among a cohort of Chinese nurses, and their knowledge of and attitudes towards occupational safety related to HIV/AIDS. Then, we measured the effectiveness of an intervention to improve nurses’ knowledge/attitudes about HIV/AIDS and HIV-related occupational exposures and universal precautions.

## 2. Methods

### 2.1. Study Setting and Participants

This study was conducted at the Affiliated Hospital of Xiangnan University located in Chenzhou, Hunan Province, a public polyclinic with more than 1000 beds. Table 1 depicts data for increased prevalence of HIV/AIDS and HIV tests for the past five years in this hospital.

All participants were clinical registered nurses working across all units (medical, surgical, pediatric, intensive care, operation room, emergency and oncology). Subjects included those with clinical roles involving patient body fluid contact or sharp medical instruments for one year in the past three years (*N* = 451). Before the intervention, during a three-day survey, all nurses on duty who satisfied inclusion criteria and voluntary were selected. After obtaining permission from the hospital management departments and mobilizing by the nursing department, a trained investigator administered 327 questionnaires to the selected nurses at their workplaces. The investigated nurses sat in separate areas to complete questionnaires anonymously and return them to the investigator on the spot. There were 300 valid questionnaires and the valid return rate was 91.7%. Interventions were carried for all nurses in the hospital. After the intervention, from the original 300 pre-intervention participants, 240 questionnaires were distributed to the post-intervention respondent nurses who finished all intervention programs and on duty during a two-day survey.

### 2.2. Study Design and Procedure

This study was conducted from October 2014 to January 2015 and included pre- and post-intervention assessments spaced three months apart. The interventions focused on occupational safety and included evaluation of intervention efficacy. A questionnaire was used to document nurses’ knowledge and attitude about HIV/AIDS-related occupational exposures before an intervention anonymously. Then, integrated interventions of multiple stages were used to deliver appropriate and targeted training at hospital, department and individual levels. After three-months of interventions, another questionnaire was given to evaluate the effectiveness of the interventions. Each questionnaire was completed independently and discussion among participants was not allowed. Prior to the official study, the questionnaire was pilot-tested on 31 nurses to determine optimal length and subject comprehension. Face validity was established by expert opinion and test-retest reliability indicated that scores for first and second questionnaires were positively correlated.

All investigated nurses gave their informed consent for study participation. The study was approved by the Institutional Review Board of XiangNan University (ECT 2013-26). All subject information was kept confidential.

### 2.3. Integrated Intervention

Study interventions were divided into five stages, which included appropriate and targeted training content for hospitals, departments or individuals. As required by management department of the hospital, all nurses should participate in intervention. The purpose of the intervention was to improve nurses’ knowledge and attitude about HIV/AIDS and HIV-related occupational exposures and universal precautionary measures.

In the first step, at the management level, administrative staff was informed about HIV/AIDS occupational exposure and improvement with universal precautions. These information sessions were provided every month to make next. Next, training classes were held for head or key nurses from departments to prepare them to educate other nurses once a month. During the intervention, three training classes were held for the nurses. The goals of these classes were to increase general knowledge about HIV/AIDS including prevention, modes of transmission such as through occupational exposure, post-exposure prophylaxis (PEP), treatment or clinical management. These classes which included lectures with discussions demonstrations and small group sessions also measured attitudes for caring for individuals with HIV/AIDS.

In the third intervention stage, peer education was performed by nurses for nurses in all departments. The head or key departmental nurses explained the knowledge learned from training courses including HIV epidemiology, modes of transmission, natural disease history, early symptoms, diagnostic testing, occupational exposure and post-exposure prophylaxis (PEP). The aim of peer education was to increase clinical nurses’ occupational safety knowledge as well as to modify attitudes towards HIV/AIDS.

In the fourth stage, the intervention was accelerated via the distribution of materials for prevention of occupational exposures to HIV, using knowledge contests (quizzes or question-and-answer games), organizing universal precautions skill competitions and with invitations to front-line staff to share experiences with carding for HIV-positive patients. Finally, the effects of the integrated interventions were assessed using *χ*^2^-test analyses.

### 2.4. Measures

The pre-intervention questionnaire included 48 questions divided into four sections. The first section collected demographic information (age, education, marital status, department, total years employed as a nurse). The second section included nine questions about occupational injuries and related outcomes. In the third section, 28 questions addressed HIV/AIDS and occupational exposure-related knowledge (six covered basic HIV/AIDS knowledge; nine covered exposure risk perception; eight were about universal precautionary protection; and five were about post-exposure prophylaxis (PEP); the fourth section included five questions focused on nurses’ attitudes about HIV/AIDS). These questions were developed after reviewing the relevant literature. All 33 questions for the third and the forth sections for the pre-intervention questionnaire were repeated in the post-intervention questionnaire. Intervention effects were assessed by improvements in knowledge and attitude based on scores to these 33 questions.

### 2.5. Data Analysis

Data were assessed with PASW Statistics 18 (SPSS Inc., Chicago, IL, USA) software. Scales were used for correct answers and a dichotomous point scale was used to measure the intervention effect on knowledge and beliefs. Correct answers before and after the intervention were compared using a chi-squared test. Mean knowledge/attitude scores were compared using an independent two-sample Student’s *t*-test. Each correct answer was one point and an incorrect answer was 0 points (*p* < 0.05 was considered statistically significant).

## 3. Results

Prior to the intervention, 300 questionnaires were collected, and, among these, 234 participants completed the program. ANOVA confirmed no significant differences in socio-demographic factors for pre-intervention and post-intervention samples. Table 2 depicts the nurses’ demographic characteristics. Table 3 depicts injuries reported and reasons for them.

Post-intervention scores confirmed that HIV/AIDS-related knowledge improved significantly and these data appear in Table 4. Nurses’ perceptions of risk of occupational exposure were high or very high and these data appear in Table 5. Nurses’ personal precaution habits for handling patient body fluids varied and these data appear in Table 6. The intervention improved personal protection awareness and correct responses increased for these five questions in the post-test. Data appear in Table 7 and Table 8. After the intervention, nurses’ attitudes about “sympathy for the suffering experienced by HIV/AIDS patients” and “do not agree that the majority of HIV/AIDS patients are suffering the consequences of their action” changed significantly (Table 8) and scores were higher after the intervention.

## 4. Discussion

Approximately 35 new HIV cases and at least 1000 serious infections are reported annually by health care workers [13,14]. HIV exposure and subsequent HIV positivity arising from health care occupational exposures appear to be relatively rare. However, contaminated NSIs represent a significant occupational burden for nurses. The prevalence of HIV/AIDS is increasing in China, and this means that hospital personnel are increasingly being exposed to hazardous body fluids from patients with HIV/AIDS as well as encountering injuries from contaminated sharp instruments. In the previous 12 months, among professional Korean nurses NSIs totaled 1.31 events/nurse/year). Table 3 depicts data regarding injuries from medical sharp instruments or splashed body fluids in the last year and the data show that nurses are at greater risk for HIV or hepatitis B and C [15].

Although 95.3% of investigated nurses in our study considered their risk of occupational exposure to be high or very high, they did fail to follow universal protection precautions. When contacting patient body fluids/blood, only 24.0% used gloves regularly. They also improperly disposed of medical waste which caused most of the occupational exposures [16]. Universal precaution protocols exist in China; however, if preventable, exposures are often accepted as being inherent to the job and not true hazards [17]. Therefore, improving knowledge of professional behaviors is essential to minimize occupational exposures to HIV.

Before our intervention, Chinese nurses did not fully understand HIV/AIDS, as the knowledge test suggested that 67.9% were correct about HIV; 54.4% gave correct answers about risk perception; 83.7% were correct about universal precautions; and 65.4% were correct about HIV post-exposure prophylaxis. Knowledge about the importance of starting post-exposure prophylaxis early is very low (28.7%). Only 30.3% have previously received occupational safety special training. The lack of awareness and related training may influence discrimination of HIV patients [18]. Thirteen percent agreed that patients with HIV/AIDS should be treated only in Infectious Diseases Hospitals. Discriminatory or unethical behavior toward patients with HIV/AIDS exists among health care professionals, as documented in other countries, which may interfere with effective prevention and treatment [19].

Effective measures to prevent nurses from occupational exposure include universal precautions, eliminating unnecessary injections, education, use of sharp instrument containers for disposal, and elimination of needle recapping, and these have reduced NSIs by 80% [12,16]. A case-control study documented that prompt initiation of zidovudine can decrease the risk of acquiring HIV by 81% after occupational exposure [20]. Post-exposure prophylaxis has come to be accepted as a standard of care for the prevention of HIV infection after a hospital-associated occupational exposure.

With the accelerating HIV epidemic in China, a well-designed educational program should be implemented as soon as possible. The integrated educational intervention implemented in this study was multifaceted, included appropriate and targeted training content, and included the hospital, the department and the individual. Specifically, these interventions included lectures with discussions, demonstrations, small group sessions, and peer education. Group discussion was necessary to decrease fear and HIV discrimination and nurses improved scores on all 33 questionnaire items about knowledge and attitudes regarding HIV-related issues, universal precautions, occupational HIV exposure and post-exposure prophylaxis, and most of them were statistically significant improvements after the intervention. The post-test knowledge scores on universal precautions were only marginally improved. Improving knowledge on universal precautions as a very important regular training program is often inspected by the Infection Control Department in this hospital. So, the pre-test scores on universal precaution were relatively high, except the item on whether the contaminated bedding and clothing should be classified for disposal.

The most important preventive measures to reduce occupational HBV (viral hepatitis type B), HIV, and HCV (viral hepatitis type C) in health care workers are education and observance of routine procedures [11]. Our integrated educational intervention enhanced nurses’ knowledge about reducing the risk of occupationally acquired HIV infection and improved observance of universal precautions, and these events decreased fear and discrimination of HIV/AIDS patients as well as increased their willingness to care for these individuals. This improved the quality of care delivered to HIV/AID patients created a novel understanding of nurses as HIV/AIDS educators in a prevention plan [11].

Limitations of the study include the single institution, the self-control before and after the intervention, and the short-term nature of the evaluation only at the individual level. Because this study uses cross-sectional convenience sampling with only self-control, results are not generalizable to other nurses in China. The short-term multifaceted HIV/AIDS educational intervention cannot allow the study of any improvements in behavior or observance of routine precautionary procedures in everyday work either. Therefore, more studies are needed to assess the effectiveness of the intervention on the reduction of risk for occupational exposure in China. Assessment on the effectiveness of the integrated intervention should be done in different hospitals and at every level including individual and administrative departments of the hospital.

## 5. Conclusions

In China, contaminated NSIs represent a significant occupational burden for nurses. However, the nurses did not fully understand HIV/AIDS, and they did fail to follow universal protection precautions. So, HIV/AIDS-related educational programs across a broader spectrum of knowledge and attitudes about HIV/AIDS in work settings are needed. The professional and integrated educational intervention will facilitate an attitude change towards HIV/AIDS and the nurses’ observance of universal precaution routine procedures in everyday work, in addition to increasing knowledge about HIV/AIDS and how to reduce their risk of occupationally acquired HIV infection. This enhancement allows nurses to assume a teaching role for the prevention and management of HIV/AIDS.

## Figures and Tables

**Table 1:** HIV (Human Immunodeficiency Virus) testing in study hospital over five years.

Year	HIV Tests	HIV Diagnoses (%)
2011	5599	3 (0.05%)
2012	6297	4 (0.06%)
2013	7852	9 (0.12%)
2014	7207	14 (0.19%)
2015	14,270	27 (0.19%)

**Table 2:** Demographics of studied nurses (*n* = 300 ^a^).

Participants		*n*	%
**Gender**	Female	294	98.0
	Male	6	2.0
**Education**	Secondary specialized	3	1.0
	Associate degree	135	45.0
	Bachelor’s degree or more	162	54.0
**Title**	Nurse	135	45.0
	Senior nurse	84	28.0
	Supervisor nurse	66	22.0
	Co-chief nurse	15	5.0
**Working site**	Medical ward	89	29.7
	Surgical ward	67	22.3
	Obstetrics and gynecology	25	8.3
	Pediatrics ward	19	6.3
	Oncology ward	18	6.0
	Infectious ward	15	5.0
	Operating room	21	7.0
	Emergency room	20	6.7
	Other	26	8.7
**Work experience (years)**	1	75	25.0
	3	56	18.7
	5	63	21.0
	10	106	35.3

^a^ 240 post-test participants were chosen from 300 subjects.

**Table 3:** NSI (Needle-stick and Sharps Injuries) reported for subjects (*n* = 300).

NSI Cause	*n*	Incidence (95% CI)
Pulling needle after infusion	164	54.7% (49.3%–60.7%)
Using ampoules	161	53.7% (48.3%–60.2%)
Recycling or destroying instruments	111	37% (31.5%–42.7%)
Installing needle or extracting liquid medicine	81	27% (22.3%–31.7%)
Adding medicine while venous transfusion	37	12.3% (8.5%–16.3%)
Cooperating with others	36	12% (8.2%–16.3%)
Using skin preparation knife	28	9.3% (6.0%–13.0%)
Other types	15	5% (2.8%–7.7%)
Total injured	283	94.3% (91.7%–96.7%)

CI: Confidence Interval.

**Table 4:** Intervention effect on HIV/AIDS knowledge.

Knowledge about HIV/AIDS	Correct Answer Number (%)		*χ*^2^	*p*-Value
	Pre-Test (*n* = 300)	Post-Test (*n* = 234)		
1. Looking at a person is enough to tell if they have AIDS (no)	277 (92.3)	223 (95.3)	4.93	0.081
2. One can get AIDS by tooth extraction or doing facials (yes)	251 (83.7)	208 (88.9)	4.01	0.132
3. Person with AIDS virus can look and feel well (yes)	130 (43.3)	155 (66.2)	36.64	0.000 *
4. Mosquito bites can spread HIV/AIDS (no)	141 (47.0)	195 (83.3)	77.25	0.000 *
5. The antibodies can be detected in the blood after a month of HIV infection (no)	157 (52.3)	152 (65.0)	25.94	0.000 *
6. One can get AIDS by sharing plates, forks, or glasses with someone who has AIDS (no)	266 (88.7)	223 (95.3)	7.54	0.021 *
Total correct answer and rate (total answer of pretest was 300 × 6 = 1800, post-test was 234 × 6 = 1404)	1222 (67.9)	1156 (82.34)	86.04	0.000 *

* *p* < 0.05; Before intervention 4.07 ± 1.18 after intervention 4.94 ± 0.94 (*t* = 9.46, *p* = 0.00).

**Table 5:** Intervention and risk perception knowledge.

Knowledge of Occupational Safety	Correct Answers (%)		*χ*^2^	*p*-Value
	Before (*n* = 300)	After (*n* = 234)		
1. Blood, semen, vaginal secretions of HIV/AIDS are sources of occupational exposure, while other kinds of body fluids such as amniotic fluid, pleural effusion and ascites are not sources	237 (79.0)	203 (86.8)	5.45	0.02
2. Risk of infection after puncture and exposure of skin to HIV-positive blood is less than 1%	25 (8.3)	73 (31.2)	45.86	0.00
3. Risk of infection is low when a large area of intact skin contacts with HIV-positive blood and infectious body fluids	139 (46.3)	146 (48.7)	0.33	0.57
4. Risk of infection is low in the case of minor scratches or abrasions of superficial skin without bleeding by HIV-contaminated instruments	94 (31.3)	126 (53.8)	27.50	0.00
5. Risk of infection is high when superficial skin is injured, and contacts with HIV-positive blood or infectious body fluids for a long time or in a large area	273 (91.0)	220 (94.0)	1.69	0.19
6. Risk of infection is low when the epidermis is punctured by HIV-contaminated solid needles (such as surgical suture needles, etc.) without bleeding	57 (19.0)	80 (34.2)	15. 90	0.00
7. Risk of infection is low for contacting with the infected person in the incubation period after minor mucous membrane injury	81 (27.0)	111 (47.4)	23.84	0.00
8. Risk of infection is high for contacting with HIV-positive blood or infectious body fluids after skin and mucous membrane damage	284 (94.7)	226 (96.6)	1.12	0.29
9. Risk of infection is high when fresh bleeding wound is caused by deep and big needle-stick injuries after skin and mucous membrane damage (such as chapping)	278 (92.7)	217 (92.7)	0.00	0.98
Correct answers (total answer pretest was 300 × 9 = 2700, post-test was 234 × 9 = 2106)	1468 (54.4)	1402 (66.6)	73.2	0.00

**Table 6:** Intervention effect on universal precaution knowledge.

Universal Precaution Item	Correct Answers (%)		*χ*^2^	*p*-Value
	Before Intervention (*n* = 300)	After Intervention (*n* = 234)		
1. Wash hands promptly after removing gloves or contact with infective material	282 (94.0)	230 (98.3)	6.13	0.02
2. When hand skin is damaged, at least two layers of gloves should be worn	195 (65.0)	194 (82.9)	21.31	0.00
3. Ensure that patient-care equipment, supplies and linen contaminated with infective material is either discarded, or disinfected or sterilized between each patient use	271 (90.3)	219 (93.7)	1.84	0.18
4. The universal precautions include protective facilities being used to avoid direct contact with body fluids. Use no touch technique wherever possible	288 (96.0)	230 (98.3)	2.37	0.12
5. The universal precautions include safe medical waste disposal. Ensure appropriate waste handling	287 (95.7)	230 (98.3)	2.94	0.09
6. The universal precautions include sharp instruments being disposed of safely. All sharp instruments should be handled with extreme care	285 (95.0)	227 (97.0)	1.34	0.25
7. The bedding and clothing contaminated with body fluids and blood of HIV/AIDS patients should be classified for disposal	122 (40.7)	117 (50.0)	4.63	0.03
8. Wear gloves when in contact with blood, body fluids, secretions, excretions, mucous membranes and contaminated items	279 (93.0)	230 (98.3)	8.25	0.00
Total correct answer (total answer pretest was 300 × 8 = 2400, post-test was 234 × 8 = 1872)	2009 (83.71)	1667 (89.58)	25.00	0.00

**Table 7:** Intervention effect on HIV post-exposure prophylaxis knowledge.

Knowledge of Occupational Safety	Correct Answers (%)		*χ*^2^	*p*-Value
	Before Intervention (*n* = 300)	After Intervention (*n* = 234)		
1. Chemoprophylaxis is unnecessary after exposure to saliva, tears, sweat of HIV/AIDS patients	107 (35.7)	134 (57.3)	24.76	0.00
2. If necessary, chemoprophylaxis should be taken within 1–2 h after an occupational exposure to HIV/AIDS	86 (28.7)	106 (45.3)	15.79	0.00
3. In the event of HIV occupational exposure, exposure site should be emergency disinfected	294 (98.0)	230 (98.3)	0.06	0.81
4. In the event of HIV occupational exposure, serum of the exposure should be reserved for later use	280 (93.3)	225 (96.2)	2.04	0.15
5. In the event of HIV occupational exposure, experts should be organized to assess exposure in order to decide whether to take chemoprophylaxis	275 (91.7)	222 (94.9)	2.09	0.15
Correct answers (total answer pretest was 300 × 5 = 1500, post-test was 234 × 22 = 5808)	4319 (65.4)	3892 (67.0)	3.41	0.06

**Table 8:** Changes in responses to attitudinal questions toward HIV/AIDS patients after intervention.

Attitudes toward HIV/AIDS Patients	Before Intervention *n* (%) (*n* = 300)	After Intervention *n* (%) (*n* = 234)	*χ*^2^	*p*-Value
1. Sympathy for the suffering of patients with HIV/AIDS	274 (91.3)	225 (96.2)	4.99	0.02
2. Not agree that the majority of HIV/AIDS should suffer the consequences of their action	225 (75.0)	202 (86.3)	10.52	0.00
3. HIV/AIDS have the right to receive the same quality of care and respect as the patients with other diseases	292 (97.3)	222 (94.9)	2.21	0.14
4. Not agree that all HIV/AIDS should seek treatment only in Infectious Diseases Hospitals	261 (87.0)	212 (90.6)	1.68	0.19
5. Strict quarantine measures are not needed during the treatment and care of patients with HIV/AIDS	89 (29.7)	88 (37.6)	3.74	0.05

## Abbreviations

HIV	Human Immunodeficiency Virus
AIDS	Acquired immunodeficiency syndrome
UNAIDS	the Joint United Nations Programme on HIV/AIDS
WHO	World Health Organization
NSIs	needle-stick and sharps injuries
CI	Confidence Interval
HBV	Viral Hepatitis Type B
HCV	Viral Hepatitis Type C
